# Effects of field conditions on the degradation of cellulose-based and PLA nonwoven mulches

**DOI:** 10.1038/s41598-025-94686-8

**Published:** 2025-04-08

**Authors:** Paula Marasović, Michał Puchalski, Dragana Kopitar

**Affiliations:** 1 Faculty of Textile Technology, Department of Textile Design and Management, University of Zagreb, Prilaz Baruna Filipovića 28a, 10000 Zagreb, Croatia; 2 Textile Institute, Lodz University of Technology, 116 ˙Zeromskiego Street, 90-924 Lodz, Poland

**Keywords:** Cellulose fibres, PLA fibres, Nonwoven needle-punched mulch, Field conditions, FTIR, WAXD, Engineering, Environmental impact

## Abstract

The impact of the field conditions on needle-punched mulches made of cellulose fibres and PLA biopolymer during the 300 days of exposure was investigated. The study observed the degradation of nonwoven mulches during specific exposure periods (30, 90, 180 and 300 days), evaluating their mechanical, morphological and chemical properties. The impact of nonwoven mulches on soil temperature and moisture, consequently on the number of microorganisms developed beneath mulches after 300 days of exposure, were analysed and associated with obtained results complementing comprehension of nonwoven mulch degradation. The findings show that nonwoven mulches made from jute, hemp, viscose and PLA fibres change when exposed to environmental conditions (soil, sunlight, rainfall, snow, ice accumulation, air and soil temperatures, wind). The changes include alterations in colour, structure shifts and modifications in properties. The results highlight the degradation pathways of cellulose and PLA mulches, revealing that cellulose-based fibres degrade through the removal of amorphous components, leading to increased crystallinity and eventual structural breakdown. WAXD findings demonstrated that microbial and environmental factors initially enhance crystalline regions in cellulose fibres but ultimately reduce tensile strength and flexibility due to amorphous phase loss. FTIR analysis confirmed the molecular changes in cellulose chains, particularly in pectin and lignin, while SEM provided direct evidence of surface damage and fibre disintegration. Furthermore, it was found that fibre types of nonwoven mulch influence soil moisture retention and soil microbial activity due to a complex interplay of fibre composition, environmental conditions and nonwoven fabric characteristics. Comprehensive mechanical, morphological and chemical results of different types of nonwoven mulch during the 300 days of exposure to the field conditions provide valuable insights into sustainable practices for using nonwoven mulches for growing crops.

## Introduction

Nonwoven mulches are crucial in agriculture and landscaping, serving various purposes such as soil erosion control, moisture retention, and weed suppression without using herbicides and pesticides^1^. As concerns about environmental sustainability grow, interest in using alternative materials such as natural fibres and polylactic acid (PLA) biopolymers for mulch production is increasing. The performance of nonwoven mulches under field conditions depends on several factors, including the properties of the materials and the environmental conditions to which they are exposed. Understanding these factors is critical to optimise mulch performance.

Regarding sustainable practices, the biodegradability of mulches produced from natural and renewable sources is a key factor in improving soil quality and reducing waste^2^.

**Fig. 1:**
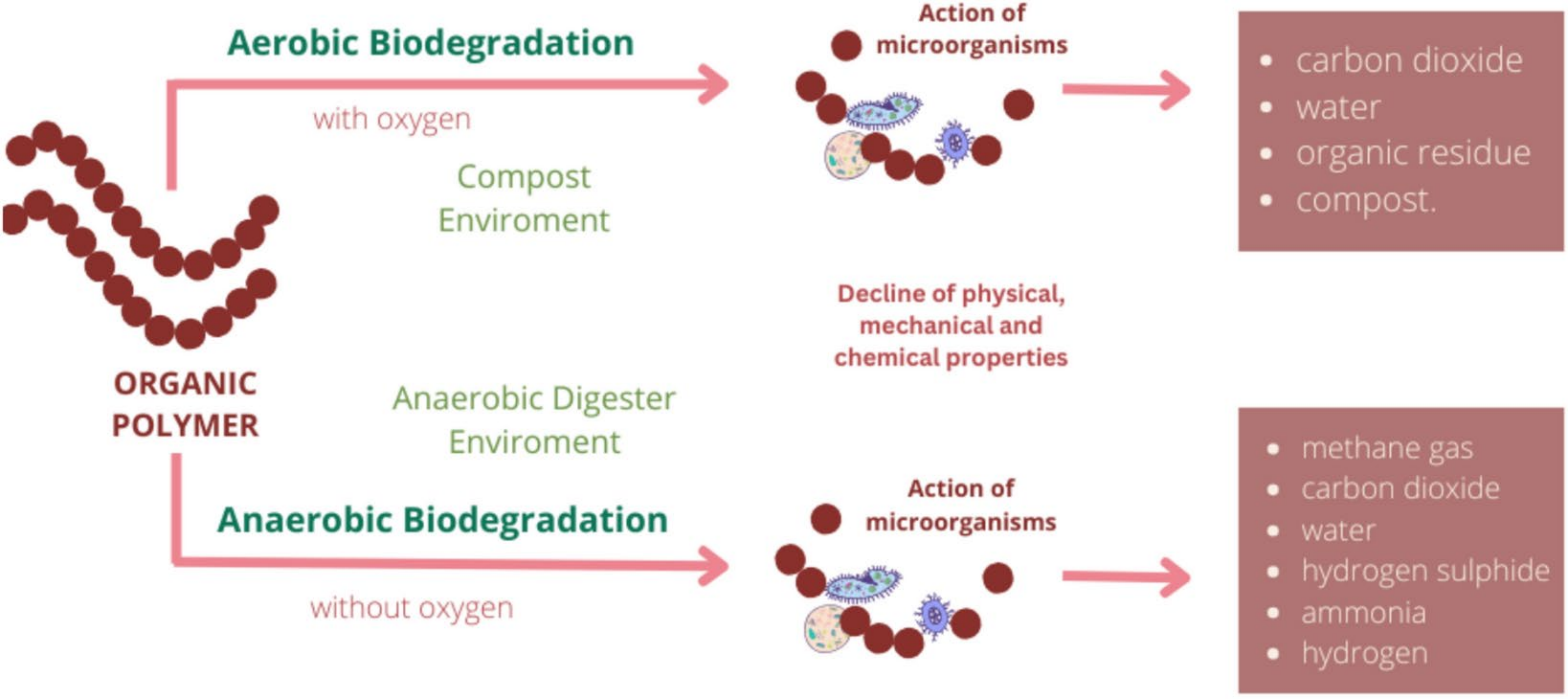
Degradation processes of organic polymers^9^.

The term biodegradation is often misinterpreted. Biodegradability refers to a polymer’s ability to decompose with the aid of living organisms into basic elements, where the polymer’s molecular weight decreases through chemical reactions like oxidation, photodegradation, and hydrolysis. Consequently, the polymer loses its physical, mechanical, and chemical properties (Fig. 1)^3,4^. Different material types and structures undergo degradation at varying rates, where factors such as temperature, moisture, oxygen availability, microbial community and soil fauna play a crucial role^5–7^. Mulches placed on the soil are gradually degraded by microorganisms, leading to mass loss, reduced mechanical properties, and the release of carbon dioxide through oxygen consumption. However, relying solely on mass loss for biodegradability assessment could lead to wrong conclusions due to the migration of water-soluble additives and polymer dissolution. Therefore, determining biodegradability, in addition to gravimetric analysis, should encompass standardised methods like tensile, respirometric, and morphological tests^8^.

As previously mentioned, researchers are exploring natural and renewable raw materials to develop biodegradable mulches as an eco-friendly alternative to reduce the adverse negative effects of plastic mulches. The degradation of biodegradable mulches is influenced by open field factors such as humidity, temperature, and UV radiation, where higher soil humidity and temperature levels accelerate the breakdown process^1^.

Research shows that nonwoven mulches made from fibres such as flax and hemp offer benefits beyond weed suppression, such as enhanced soil health, nutrient retention, waste reduction, and improved plant yield. Besides the good weed suppression, the benefits of jute nonwoven mulches are increased yield and enhanced moisture and nutrient availability in the soil compared to conventional plastic agrotextiles^1^.

Viscose fibre is gaining popularity for mulching and seedlings due to its excellent absorption capacity and relatively fast biodegradation. Viscose nonwoven mulches enhance soil moisture retention, making them especially valuable in arid regions where conserving water is vital for crop growth^10–13^. Viscose fibres are composed solely of cellulose, making them highly hygroscopic and prone to moisture absorption. Its lower polymerisation degree, weaker molecular orientation, and reduced crystallinity cause swelling and stiffening when wet, limiting its biodegradability.

Microbial enzymatic degradation of cellulose fibres mainly involves bacteria and fungi, with degradation initiating from the surface and progressing inward. Microorganisms break the secondary wall to multiply within the lumen after breaking the cuticle. With microorganisms multiplying, the biodegradation process advances, targeting amorphous regions of the fibres first. Therefore, fibres with lower crystallinity display higher biodegradation rates since the amorphous regions decompose before crystalline ones^14–17^.

Polylactic acid (PLA) polymer is emerging as a promising material for biodegradable agrotextiles products. Research on the biodegradation of PLA agro textiles in various environments has found that degradation is a complex process, exhibiting distinct patterns of morphological changes. The mechanism of PLA degradation includes hydrolysis, catalysed by temperature, followed by bacterial decomposition of the residues^18^. Temperature is a key factor in PLA degradation, with most studies reporting biodegradation occurring efficiently at high temperatures, around 58 °C. In industrial composting systems (55–65 °C), PLA breaks down within 45 to 60 days, while in natural soil, the process is much slower, often taking several months. Laboratory simulations of aerobic composting have shown complete degradation of PLA nonwoven agrotextiles within 16 weeks, with crystallinity variations (11.1–33.6%) having little effect. Microbial activity is crucial, accelerating degradation in warm composting conditions while remaining minimal or absent at lower temperatures (25–37 °C)^6,8,19–22^. Real soil environments often do not exceed temperatures of 30 °C, meaning that degradation of PLA nonwoven mulch exposed to field conditions is challenging^18^.

A potential strategy (if needed) to prevent cellulose fiber degradation could involve optimising the fiber composition and structural properties. For instance, incorporating PLA into cellulose fibers may enhance their resistance to degradation by forming a protective matrix that slows microbial breakdown without the need for non-ecological additives. Additionally, to slow down degradation, PLA can be made in various composites to extend its lifespan. PLA’s mechanical properties can be enhanced by incorporating cellulose nanocrystals (CNC), improving ductility, tensile strength, and thermal resistance^23^.

This comparative study investigated the influence of field conditions on nonwoven needle-punched mulches made of cellulose fibres (jute, hemp, and viscose fibres) and PLA biopolymer during 300 days of open-field exposure. The degradation processes of the mulches were assessed by examining their physical–mechanical, morphological, and chemical properties during specific time intervals. The impact of nonwoven mulches on soil temperature and humidity is analysed during the degradation of mulches on the field. The influence of mulch degradation on the soil was determined by comparing the number of microorganisms in the soil after 300 days of mulching with different mulch types and control fields.

By comprehensively understanding the interplay between biodegradation and the structural and mechanical properties of researched mulches, the study offers valuable insights into sustainable mulching practices and their efficacy under environmental conditions. This research significantly contributes to understanding sustainable mulching techniques performed in field conditions.

## Materials characterisation

The nonwoven mulches are made from regenerated viscose, jute, hemp (Derotex NA), and PLA fibres (NatureWorks LLC) in nominal mass per unit area of 400 g m^−2^. Mulches were produced using the same technological line with the same production parameters on the card and bonded by needling^24^. The experiment was conducted in Croatia (45°53′ N, 15°44′ E), in a region characterised by a humid continental climate (Dfa) based on the Köppen–Geiger climate classification. Each mulch of dimension 1.5 m × 1.5 m (2.25 m^2^) was randomly distributed across the soil in blocks of four replicated plots. Each block included a control field (bare, uncovered soil). The experiment began in April 2022 and finished in February 2023, when the last replication plot of nonwoven mulches was collected. After certain time intervals (30, 90, 180 and 300 days) of exposure in the open field, one plot of nonwoven mulch was collected and evaluated. The degradation of mulch was evaluated through visual inspection, SEM analysis, physical–mechanical tests, FTIR analysis, and WAXD analysis. Soil temperature and moisture beneath the mulches and on the control field were monitored weekly. Before the experiment and after 300 days of exposure, soil samples beneath each mulch type and on the control field were collected and analysed for microbial activity.

## Methods

### Scanning electron microscope (SEM) analysis

After each mulch plot was removed from the field, detailed physical features of the mulches were analysed on a microscale using a scanning electron microscope (SEM). Microscopic examination of exposed nonwoven mulches was performed with the Nova NanoSEM 230 system from FEI Company (Eindhoven, The Netherlands) by applying parameters such as SE Detector: Secondary Electron, High Voltage: 10 kV, Low Vacuum: 0.63 Torr, and Magnification: × 800.

### Mass per unit area and thickness

The mass per unit area of nonwoven mulch is determined following the ISO 9073–1:2023 standard for nonwoven fabrics. Five samples, each dimension of 350 × 200 mm, were weighed on an analytical balance (precision of ± 0.0001 g). The mass of the nonwoven fabrics was used to calculate the mass per unit area (g/m^2^), expressed per square meter.

The thickness of the nonwoven mulch is measured following ISO 9073–2:1995 using a pressure of 0.5 kPa. Ten samples of each type were prepared and tested using a thickness gauge from Schmidt Technology GmbH (Georgen, Germany).

### Tensile properties

The breaking force and elongation at the break of nonwoven mulches were evaluated following ISO 9073–3:2023 (wide strip method). Five samples per mulch type, with dimensions of 350 mm × 200 mm, were tested in both the machine direction (MD) and cross-machine direction (CD) using the Tenso Lab 5000 tensile tester (Mesdan S.p.A). Testing was performed at a constant speed of 100 mm/min with a pre-tension of 5 N.

### Fourier-transform infrared (FTIR) analyses

The FTIR analyses were conducted on unexposed mulches and mulches exposed for specific periods. Examinations utilised an FTIR spectrometer (PerkinElmer Spectrum One, MA, USA) at room temperature and humidity. Solid samples were placed in their natural state on the ATR crystal (Zn/Se crystal) to maintain measurement accuracy and reliability. Spectral data were recorded within the range of 4000 cm^−1^ to 650 cm^−1^, with a spectral resolution of 4 cm^−1^, allowing for an accurate and comprehensive analysis of molecular vibrations and chemical characteristics.

### Wide-angle X-ray diffraction method

The crystalline structure of nonwoven samples was characterised using an X’Pert PRO diffractometer (PANalytical, Almelo, The Netherlands) with a CuKa source (λ = 0.154 nm) with an accelerating voltage of 40 kV and an anode current density of 30 mA. A semiconductor counter X’Celector was used as the detector. The diffraction patterns for the powdered samples were recorded over a 2ϴ range of 5–50° ° with a step size of 0.015. The degree of crystallinity was estimated using WAXSFIT and Hindeleh and Johnson’s method according to the following equation:

1 $${\text{x}}_{{\text{o}}} { = }\frac{{{\text{A}}_{{\text{C}}} {\text{ + A}}_{{\text{M}}} }}{{{\text{A}}{}_{{\text{A}}}{\text{ + A}}_{{\text{C}}} {\text{ + A}}_{{\text{M}}} }}100\%$$

where A_A_, A_C_ and A_M_ are the integral intensities of the amorphous halo and the peaks originating from the crystalline phase and, if present, meso-phase, respectively.

The characteristic feature of the nonwoven mulch supramolecular structures after exposure to the field conditions was detected by analysis of the d-spacing (lattice length), estimated using Bragg’s equation:

2 $${\text{d}} = \frac{{\uplambda }}{{2\sin {\uptheta }}}$$

where $\lambda$ is the wavelength of the X-ray source (0.15418 nm), and ϴ is the angle of the reflection (half of 2ϴ of the peak position).

The crystalline area size corresponding to the (hkl) lattice planes was estimated using Scherrer’s formula:

3 $${\text{L}}_{{({\text{hkl}})}} = \frac{{{\text{k}}\lambda }}{{\beta {\text{cos}}\theta }}$$

where L_(hkl)_ is the average size of the crystalline area corresponding to the (hkl) lattice planes, λ is the Bragg angle for (hkl) planes, λ is the wavelength of X-ray radiation (for CuKa α λ = 0.15418 nm), β full width at half maximum of the diffraction peak in radians for (hkl) planes and K is the Scherrer constant (usually taken as about 0.9 for polymers, as well as in this case).

### Soil temperature and moisture

Soil moisture at a depth of 15 cm beneath the mulches and on the control field (bare, uncovered soil) was measured using a PMS-714 soil moisture meter (Lutron Electronic Enterprise Co., Ltd., Taipei, Taiwan). Soil temperature at the same depth was recorded with a bi-metal dial thermometer featuring a long, waterproof stainless-steel probe from LabTherm XL (Dostmann electronic GmbH, Wertheim-Reicholzheim, Germany). Air temperature and humidity during the mulches’ exposure to open field conditions were monitored and obtained from the nearest hydrometeorological station.

### Number of microorganisms (Bacteria and Fungi) in soil

The population of bacteria and fungi in the soil beneath the mulches and on the control field was evaluated through standard microbiological methods. Soil samples were homogenised in a sterile saline solution for five minutes. Nutrient agar (NA) was employed to measure bacterial populations, while Czapek agar was used to assess fungal populations. Each sample underwent triplicate analysis. After incubation, the colonies formed on the media were counted, and the CFU (colony-forming unit) values per gram of soil were calculated for each type of microorganism.

## Results and discussion

### Nonwoven mulches visual inspection and SEM analysis

**Fig. 2:**
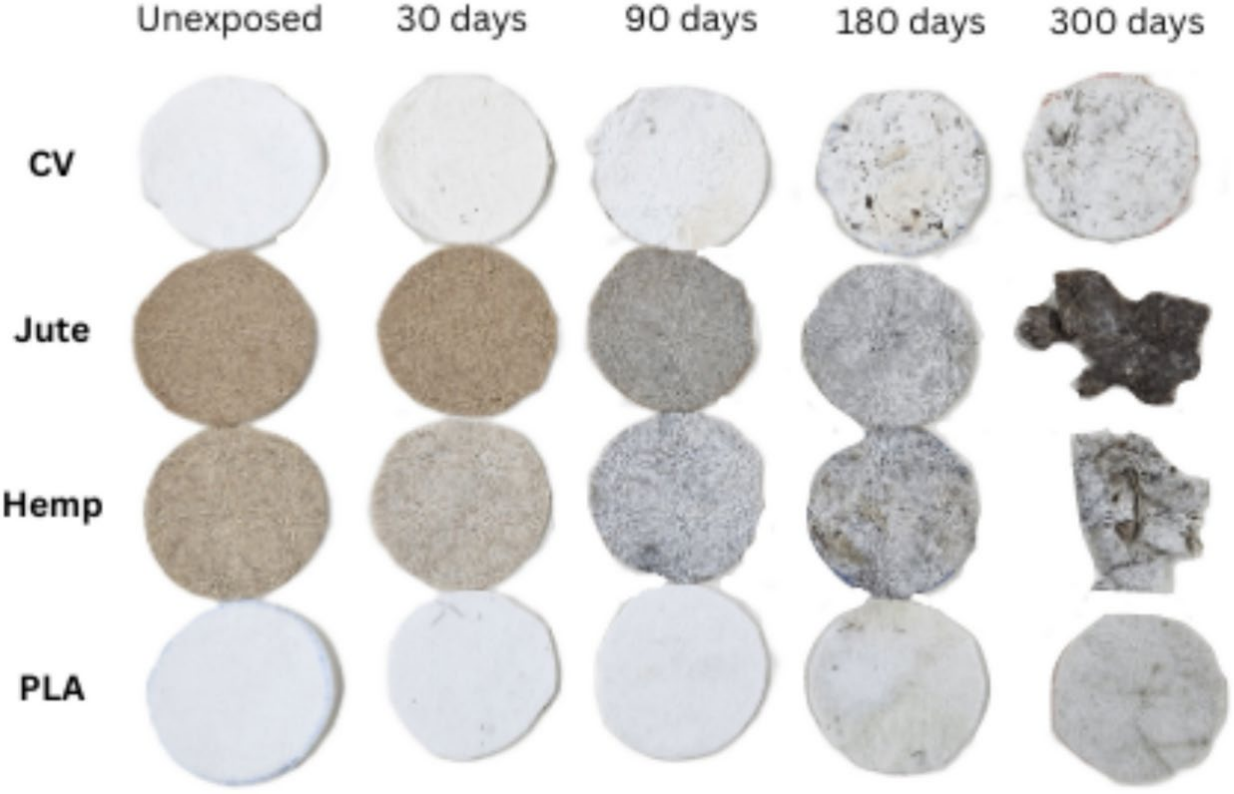
Unexposed nonwoven mulch (0 days) and mulch exposed to the field conditions for 300 days.

Visual inspection of nonwoven samples exposed to outdoor conditions reveals changes in their appearance (Fig. 2). All nonwoven samples exhibit a colour change, respectively; exposure to the soil, sunlight, and precipitation caused nonwoven mulch discolouration. The colour change is particularly pronounced in nonwoven mulch with lignocellulosic fibres, i.e. jute and hemp.

Lignocellulosic fibres should contain 2.5% or more lignin to begin photo-yellowing and lose colour fastness. The UV radiation causes the phenolic hydroxyl groups in the lignin to change into quinoid structures and produce free radicals. Due to the surface yellowing caused by UV radiation, raw bast fibres faded significantly^25^.

The impact of field exposure extends beyond mere discolouration. Nonwoven mulch made from viscose fibres undergoes a significant shift in texture, transitioning from a soft, fluffy structure to a condensed and paper-like. The structure changes suggest that viscose fibres react to environmental factors, especially moisture, causing compressed and hardened mulch structures over time.

Dimensional changes in nonwoven mulches during the exposure period were notable. Shrinkage and expansion of mulches occurred due to environmental influences such as rain, dew, soil, humidity, and sunlight. These changes followed no consistent pattern. After 180 days under field conditions, viscose mulches experienced a 3.3% shrinkage in the machine direction (MD) and a 3.3% expansion in the cross-machine direction (CD). By 300 days, compared to unexposed samples, viscose mulches had shrunk by 6.1% in the MD and 7.4% in the CD. Nonwoven mulches made from jute and hemp fibres displayed similar trends, with expansion in the MD direction (4.7%, 0.7%) and shrinkage in the CD direction (2.7%, 1.0%) after 180 days. After 300 days of exposure, jute mulches showed more remarkable dimensional changes, whereas hemp mulches returned to nearly their original size just before fully degrading. Mulches made of PLA fibres exhibited variability in dimensional changes, which remained below 2.0%.

**Fig. 3:**
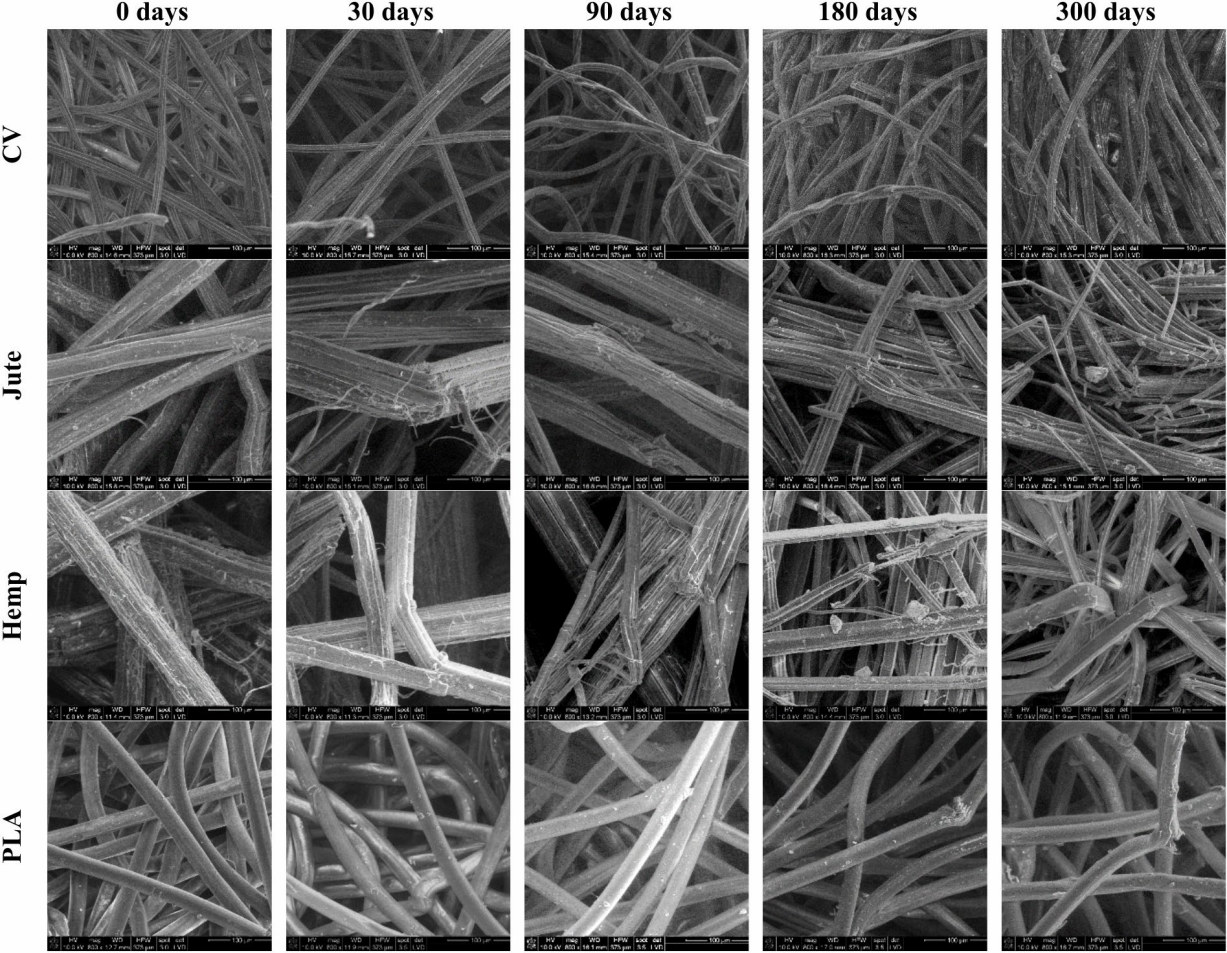
SEM images of nonwoven mulch surface before and after exposure to the field conditions in specific periods.

The SEM images show that regenerated viscose, jute, hemp, and PLA fibres of exposed nonwoven mulches (Fig. 3) exhibit surface damage, roughness, and noticeable cracks compared to the unexposed mulches. Viscose fibre, composed entirely of cellulose, has hygroscopic properties, which means it absorbs moisture from its surroundings. Due to their low polymerisation degree, weaker molecular alignment, and reduced crystallinity, the fibres swell and become stiffer when exposed to moisture. Unexposed viscose fibres exhibit distinctive longitudinal grooves on their surface, resulting from the chemical spinning process during filament solidification and pulp regeneration, specifically surface wrinkling. Viscose fibres from exposed mulches exhibit increased wrinkling during exposure to field conditions. Additionally, after 90 and 180 days of exposure, the “protective skin" formed during the spinning process of viscose fibres starts to wrinkle in some areas. After 300 days of exposure to the open field, multiple defects appear on the fibre surface, and more pronounced surface cracking and scars can be observed. The regenerated viscose nonwoven mulch contains some amount of cotton fibre, which probably arose during the production of nonwoven fabrics on the line where cotton fibres were previously processed.

The degradation of exposed mulches is particularly pronounced in hemp and jute samples, as their higher moisture absorption capacity fosters favourable conditions for microbial growth. Images of hemp and jute fibres from exposed nonwoven mulches show surface damage, roughness, and significant cracks compared to fibres from unexposed mulch. The hemp and jute fibres comprise multiple elementary fibres held together by internal pectin. The pectin and lignin serve as a protective layer, but at the same time, pectin is a preferred food source for microorganisms. The images suggest that the degradation of hemp fibres began first, which is expected since hemp fibres contain less lignin and pectin than jute fibres. Images show that the internal pectin in hemp fibres started to expose elementary fibres after 90 days in the open field. After 300 days, the interior pectin in hemp fibres was attacked by microorganisms, resulting in an image with predominantly elementary fibres.

Before exposure to open field conditions, the PLA fibres of nonwoven mulches had smooth and clean surfaces. However, after the mulches were exposed to open field conditions for 90 days, dirt and several cracks were evident on the mulches’ fibre surfaces. Extending the exposure period to 180 and 300 days, cracks on fibres became intensive. At the end of the experiment, the surface of the PLA fibres became rougher, and cracks increased due to exposure to field conditions.

### Mass per unit area and thickness of the nonwoven mulches

**Fig. 4:**
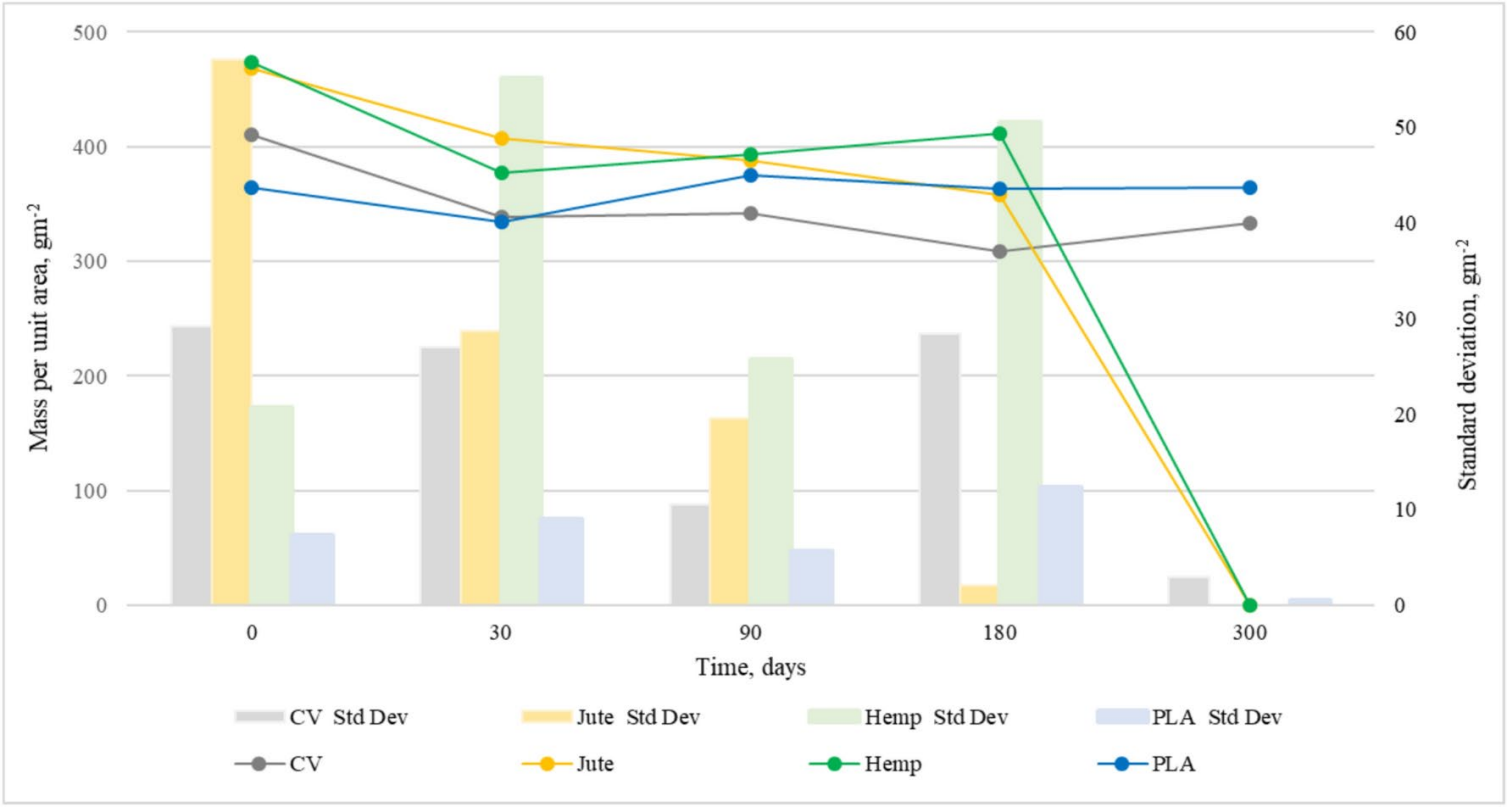
Variation of nonwoven mulch mass per unit area during 300 days of field exposure conditions.

**Fig. 5:**
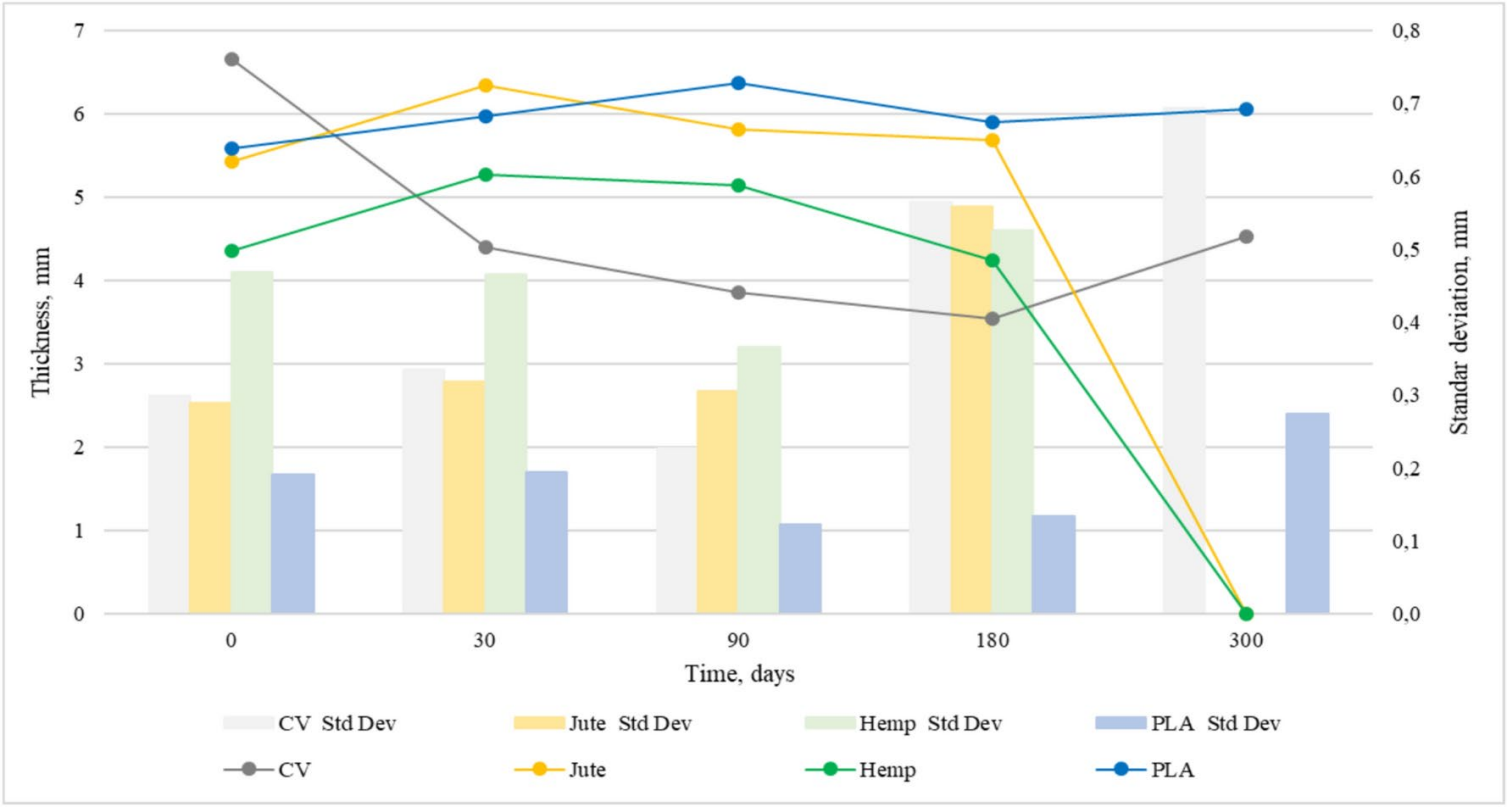
Variation of nonwoven mulch thickness during 300 days of field exposure conditions.

Figures 4 and 5 show the changes in mass per unit area and thickness of the nonwoven mulches during exposure to field conditions. The mass per unit area and thickness of nonwoven mulches increases and decreases during the exposure period, i.e. it doesn’t decrease linearly as expected.

After 30 days of exposure, the mass per unit area of jute, hemp, and PLA mulches decreases while thickness increases. Considering the change in mulch dimensions (Table 1), it is evident that mulch shrinkage and expansion influence mulch mass and thickness. As previously noticed, mulches of viscose fibres change their structure from soft and fluffy to condensed and paper-like. Therefore, only viscose mulches have a decrease in both mass and thickness.

**Table 1:** Change in dimension (%) of nonwoven mulches after exposure to the open field.

Days	180		300	
Mulch production direction	MD	CD	MD	CD
CV	− 3.3	+ 3.3	− 6.3	− 0.7
Jute	+ 4.7	− 2.7	D	D
Hemp	+ 0.7	− 1.0	D	D
PLA	− 1.7	+ 1.7	+ 1.0	+ 1.5

Where D denotes degraded mulch, MD change in the dimension of nonwoven fabric in the machine direction, and CD change in the dimension of nonwoven fabric in the cross-machine direction.

At 90 and 180 days of exposure, mulches exhibit a decrease and increase of mass and thickness as an outcome of dimensional mulches change. After 300 days of exposure, jute and hemp mulch completely degraded. After 180 days, the mass and thickness of viscose mulch stop decreasing and, on 300 days of exposure, increase. Changes in PLA mulch’s mass and thickness are not significant. It is assumed that, in addition to dimensional mulch changes, fluctuations of mass and thickness values are also influenced by impurities entangled in the material. Therefore, mulch mass per unit area and thickness cannot be reliable and solely indicators of mulch degradation.

Statistical data of mass per unit area and thickness are presented in Table 2, revealing clear trends in material degradation over time. The nonwoven mulches mass per unit area and thickness showing notable reductions, particularly for cellulose-based materials. The nonwoven mulches coefficient of variation (CV%) of mass per unit area and thickness remains relatively low for PLA across all time points, confirming its stability. The higher variation coefficient values for viscose nonwoven mulces at 180 and 300 days of field exposure suggest uneven degradation. The statistical error for mulches’ mass per unit area and thickness is generally low, reinforcing the reliability of the measurements. The increased SE values at later stages of field exposure for some mulches reflect greater measurement dispersion due to degradation effects.

**Table 2:** Statistical data of nonwoven mulches for mass per unit area and thickness.

		**Mass per unit area (g m**^**−2**^**)**					**Thickness (mm)**				
		**0**	**30**	**90**	**180**	**300**	**0**	**30**	**90**	**180**	**300**
CV	X̄	410.79	338.11	342.37	308.84	333.22	6.66	4.41	3.85	3.54	4.53
	SD	29.24	27.05	10.59	28.44	2.95	0.30	0.33	0.23	0.57	0.69
	CV, %	7.12	8.00	3.09	9.21	0.89	4.50	7.59	5.91	15.98	15.33
	SE	13.08	12.10	4.74	12.72	1.32	0.09	0.11	0.07	0.18	0.22
Jute	X̄	468.80	407.49	387.81	358.15	D	5.43	6.34	5.82	5.69	D
	SD	57.11	28.68	19.50	2.08	D	0.29	0.32	0.31	0.56	D
	CV, %	12.18	7.04	5.03	0.58	D	5.33	5.03	5.24	9.82	D
	SE	25.54	12.83	8.72	0.93	D	0.09	0.10	0.10	0.18	D
Hemp	X̄	473.41	377.47	392.96	411.06	D	4.35	5.27	5.14	4.25	D
	SD	20.69	55.14	25.67	50.56	D	0.47	0.46	0.36	0.53	D
	CV, %	4.37	14.61	6.53	12.30	D	10.76	8.83	7.10	12.40	D
	SE	9.25	24.66	11.48	22.61	D	0.15	0.15	0.12	0.17	D
PLA	X̄	363.91	333.88	374.76	363.77	364.85	5.59	5.97	6.37	5.90	6.05
	SD	7.29	8.96	5.61	12.35	0.58	0.19	0.19	0.12	0.13	0.27
	CV, %	2.00	2.68	1.50	3.39	0.16	3.41	3.26	1.93	2.28	4.52
	SE	3.26	4.01	2.51	5.52	0.26	0.06	0.06	0.04	0.04	0.09

X̄ is average mass per unit are (g m^−2^) or thickness (mm), SD is standard deviation of mass per unit are (g m^−2^) or thickness (mm), CV is coefficient of variation in %, SE is standard error of mass per unit are (gm^−2^) or thickness (mm), D stands for degraded.

### Tensile properties of the nonwoven mulches

**Fig. 6:**
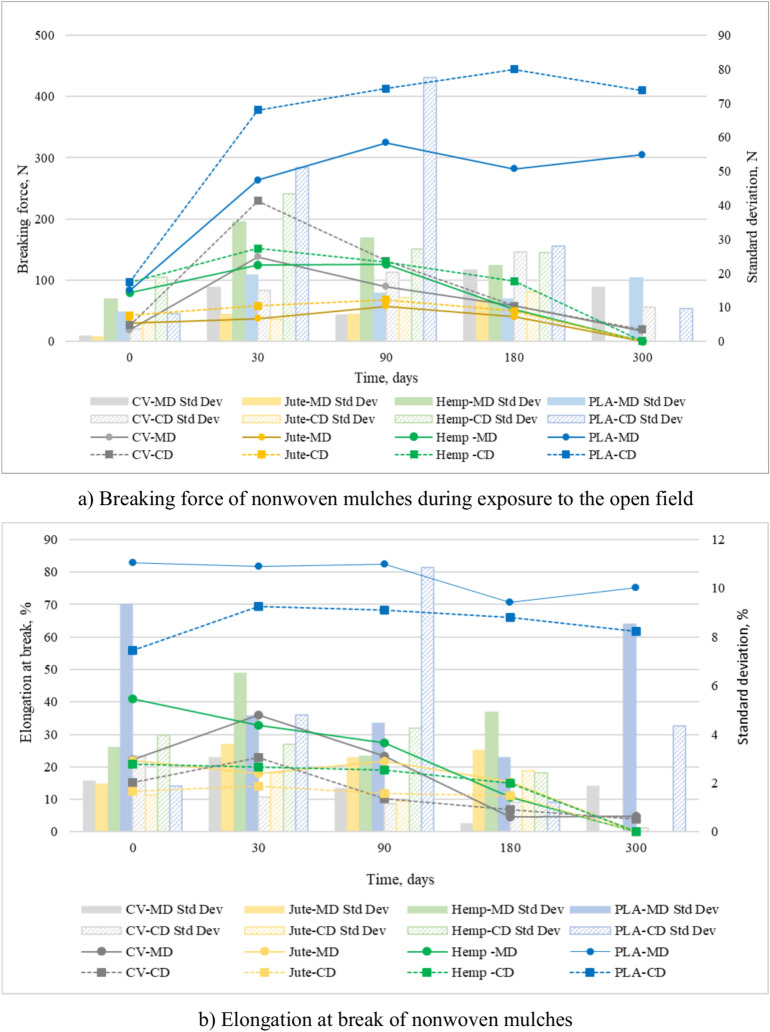
(**a**) Breaking force and (**b**) Elongation at break of nonwoven mulches in both production directions (MD and CD) during exposure to the field conditions.

After 30 days on the open field, all nonwoven mulches exhibit a notable increase in breaking force in both production directions (Fig. 6a). As previously discussed, the structural changes of the nonwoven mulches impacted the samples’ breaking force in the MD and CD directions. Factors such as fibre strength, arrangement, flexibility, diameter, and surface texture influence breaking force, with open-field conditions playing a crucial role. Additionally, changes in mulch dimension, such as shrinkage and expansion, affect breaking force. In cases where the mulch predominantly shrinks, more fibres per unit area establish mutual bonds, stiffening the material and increasing the breaking force.

Viscose mulches show the most significant structural changes after 30 days of exposure, followed by PLA mulches, while hemp and jute mulches exhibit the least changes. Accordingly, viscose mulches show the highest increase in breaking force in both production directions (in MD from 18.66 N to 138.10 N; in CD from 26.24 N to 229.50 N) after 30 days of exposure. In the same exposure period, hemp (MD from 79.10 N to 124.20 N; CD from 96.20 N to 151.90 N) and jute (MD from 29.90 N to 36.80 N; CD from 42.50 N to 57.60 N) mulches show the least increase in braking force. The breaking force continues to increase and/or decrease during the following days of open field exposure.

Exposure of viscose mulches to open field conditions over 300 days led to a decrease in mass per unit area and thickness and deterioration of viscose fibres, consequently decreasing the mulch’s breaking force. At the end of the experiment, the jute and hemp mulches deteriorated significantly, and they could not be collected from the field for tensile testing. Unlike mulches made of viscose and bast fibres, the breaking force of mulches made of PLA fibres mainly increases until the end of the experiment. At the end of the experiment, the breaking force of PLA mulches (MD 304.94 N; CD 410.08 N) was significantly more significant than the breaking force of unexposed PLA mulch (MD 83.16 N; CD 96.88 N). As previously commented, exposure of PLA mulches in the open field increased mass per unit area and thickness; the PLA fibres within the mulch came closer together, consequently increasing the mulch’s breaking force.

During the exposure period, the elongation at break of mulches made of viscose and jute fibres increases after 30 days of exposure in the MD and CD direction, after which it decreases until the last day of the experiment. Hemp mulches have a linear decrease in elongation at the break during exposure to open field conditions in both production directions. Unlike cellulose mulches, the elongation at break of PLA mulches increases and decreases over exposure time (up to 24% of the absolute value) without showing any discernible patterns (Fig. 6b).

Based on previous observations regarding mulch shrinkage, the stiffening effect resulting from this process leads to a decrease in the material’s pliability and elongation. This decrease in elongation is evident in cellulose mulches. However, mulches made of PLA fibres do not exhibit this trend, as they are less affected by field conditions and maintain their elongation more effectively over time, even when exposed for 300 days. The statistical data in Table 3 shows that PLA has low variability and consistent performance in breaking force and elongation over 300 days, indicating stability. However, cellulose-based mulches show increased variability, particularly in the later stages of field exposure, indicating mulches’ degradation. Elongation at break of mulches decreases over time, with CV mulch showing increased variability, indicating reduced ductility and instability.

**Table 3:** Statistical data of nonwoven mulches for tensile properties.

		Breaking force-MD (N)					Breaking force-CD (N)				
		0	30	90	180	300	0	30	90	180	300
CV	X̄	18.66	138.10	89.50	57.96	16.80	26.24	229.50	131.40	57.98	19.10
	SD	1.57	15.80	7.60	20.88	15.74	3.57	15.00	20.30	26.24	10.05
	CV, %	8.43	11.44	8.49	36.02	93.72	13.61	6.54	15.42	45.25	52.60
	SE	0.70	7.07	3.40	9.34	7.04	1.60	6.71	9.08	11.73	4.49
Jute	X̄	29.90	36.80	57.20	39.78	D	42.50	57.60	68.10	49.28	D
	SD	1.20	7.80	7.90	12.21	D	5.70	6.50	12.80	14.47	D
	CV, %	4.16	21.23	13.82	30.69	D	134.02	11.36	18.78	29.36	D
	SE	0.54	3.49	3.53	5.46	D	2.55	2.91	5.72	6.47	D
Hemp	X̄	79.10	124.20	125.62	52.24	D	96.20	151.90	129.86	98.00	D
	SD	12.40	35.00	30.37	22.21	D	18.90	43.30	27.13	26.05	D
	CV, %	15.67	28.21	24.17	42.52	D	19.69	28.49	20.90	26.59	D
	SE	5.55	15.65	13.58	9.93	D	8.45	19.36	12.13	11.65	D
PLA	X̄	83.16	263.64	324.80	281.58	304.94	96.88	377.94	412.82	444.76	410.08
	SD	8.53	19.40	14.08	12.42	18.58	8.12	51.03	77.58	27.96	9.57
	CV, %	10.26	7.36	4.33	4.41	6.09	8.38	13.50	18.79	6.29	2.33
	SE	3.82	8.68	6.29	5.55	8.31	3.63	22.82	34.70	12.50	4.28

		Elongation at break-MD (%)					Elongation at break-CD (%)				
		0	30	90	180	300	0	30	90	180	300
CV	X̄	22.27	36.08	23.26	4.68	4.89	15.13	22.85	10.31	6.94	4.00
	SD	2.07	3.04	1.78	0.34	1.87	3.11	1.42	1.14	1.20	0.16
	CV, %	9.31	8.42	7.67	7.36	38.24	20.53	6.23	11.04	17.34	4.00
	SE	0.93	1.36	0.80	0.15	0.84	1.39	0.64	0.51	0.54	0.07
Jute	X̄	21.99	17.91	21.65	15.34	D	12.44	13.99	11.92	11.09	D
	SD	1.97	3.59	3.06	3.36	D	1.52	2.41	1.26	2.50	D
	CV, %	8.98	20.03	14.12	21.91	D	12.21	17.25	10.56	22.55	D
	SE	0.88	1.61	1.37	1.50	D	0.68	1.08	0.56	1.12	D
Hemp	X̄	40.85	32.87	27.50	10.60	D	20.84	19.85	19.08	14.89	D
	SD	3.46	6.50	3.11	4.91	D	3.95	3.60	4.24	2.42	D
	CV, %	8.46	19.77	11.31	46.30	D	18.94	18.12	22.24	16.25	D
	SE	1.55	2.91	1.39	2.20	D	1.77	1.61	1.90	1.08	D
PLA	X̄	82.78	81.72	82.50	70.71	75.23	55.92	69.45	68.19	66.01	61.74
	SD	9.35	4.76	4.46	3.05	8.54	1.87	4.80	10.86	1.22	4.34
	CV, %	11.30	5.82	5.41	4.32	11.35	3.35	6.92	15.93	1.84	7.04
	SE	4.18	2.13	1.99	1.37	3.82	0.84	2.15	4.86	0.54	1.94

X̄ is average breaking force (N) or elongation at break (%), SD is standard deviation of breaking force (N) or elongation at break (%), CV is coefficient of variation in %, SE is standard error of breaking force (N) or elongation at break (%), D stands for degraded.

The results of nonwoven mulches’ mass per unit area, thickness, and tensile properties do not provide reasonable and clear evidence of mulch degradation during 300 days of exposure to open field conditions. The reason could be found in changes in the mulches on two levels: first, a change in the layered structure of nonwoven mulches due to environmental factors (dimensional changes, mass per unit area, thickness) and second, fibre changes (deterioration), especially noted for mulches produced from cellulose fibres. It can be concluded that the mentioned parameters can not be degradation indicators for mulches produced on cards and bonded by needling. To gain detailed information about fibre degradation within the exposed mulches during open field exposure, FTIR and WAXD analyses of the fibres within mulches were performed.

### Fourier-transform infrared analyses

**Fig. 7:**
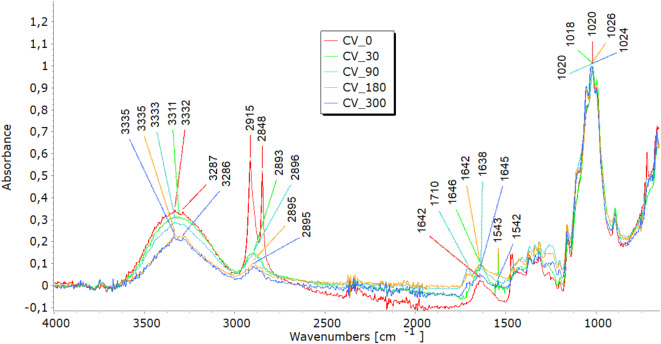

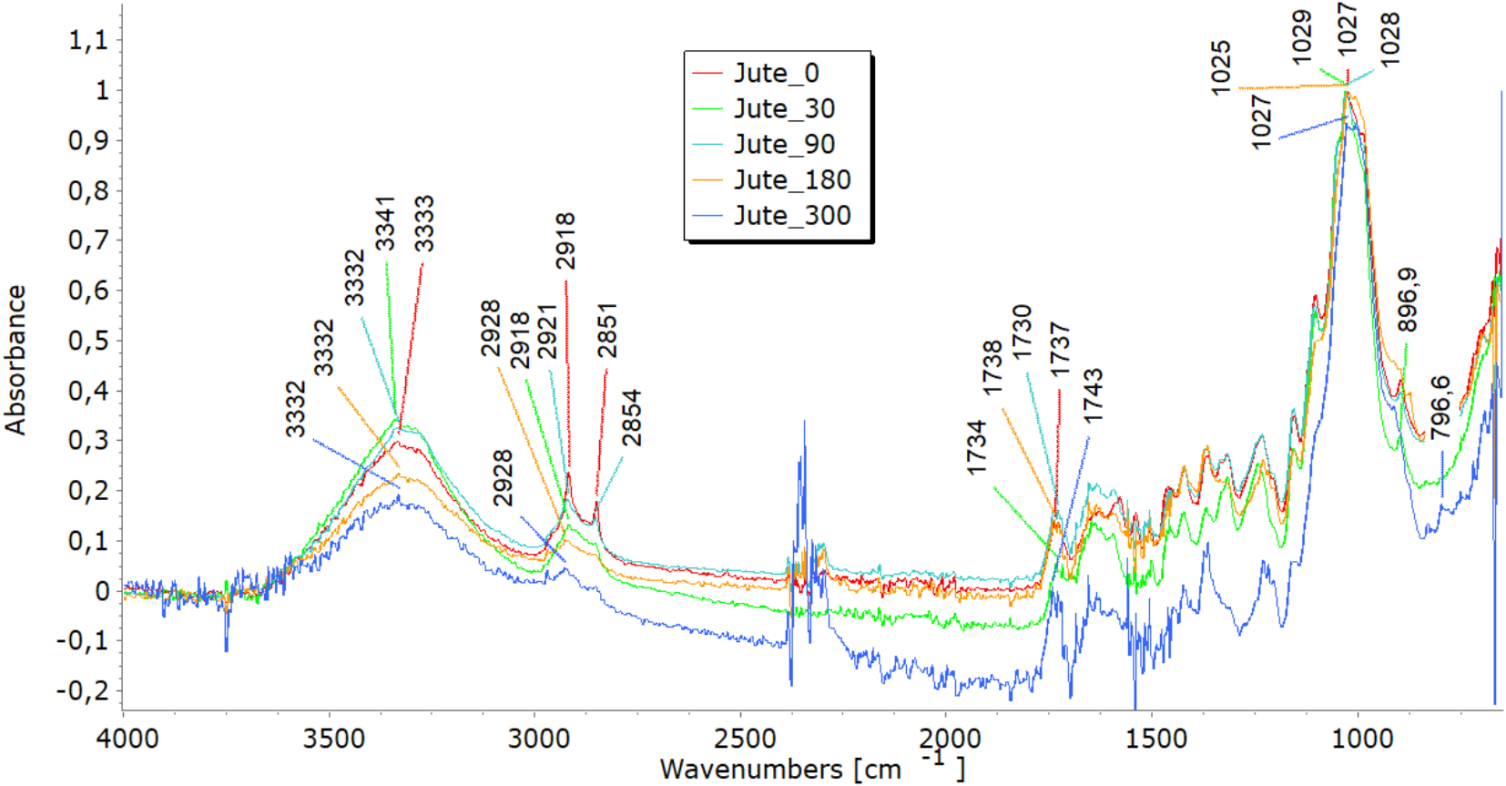

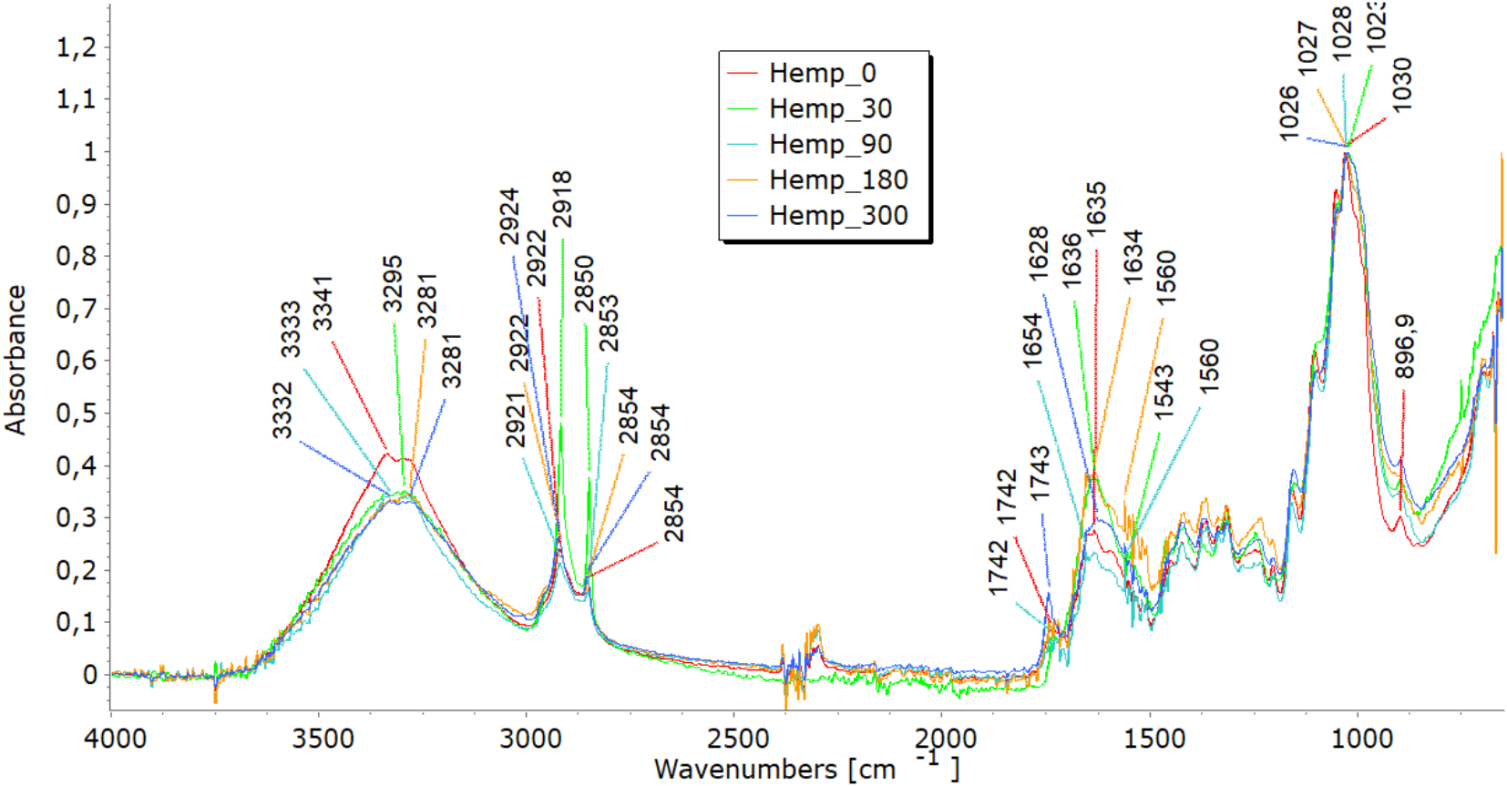

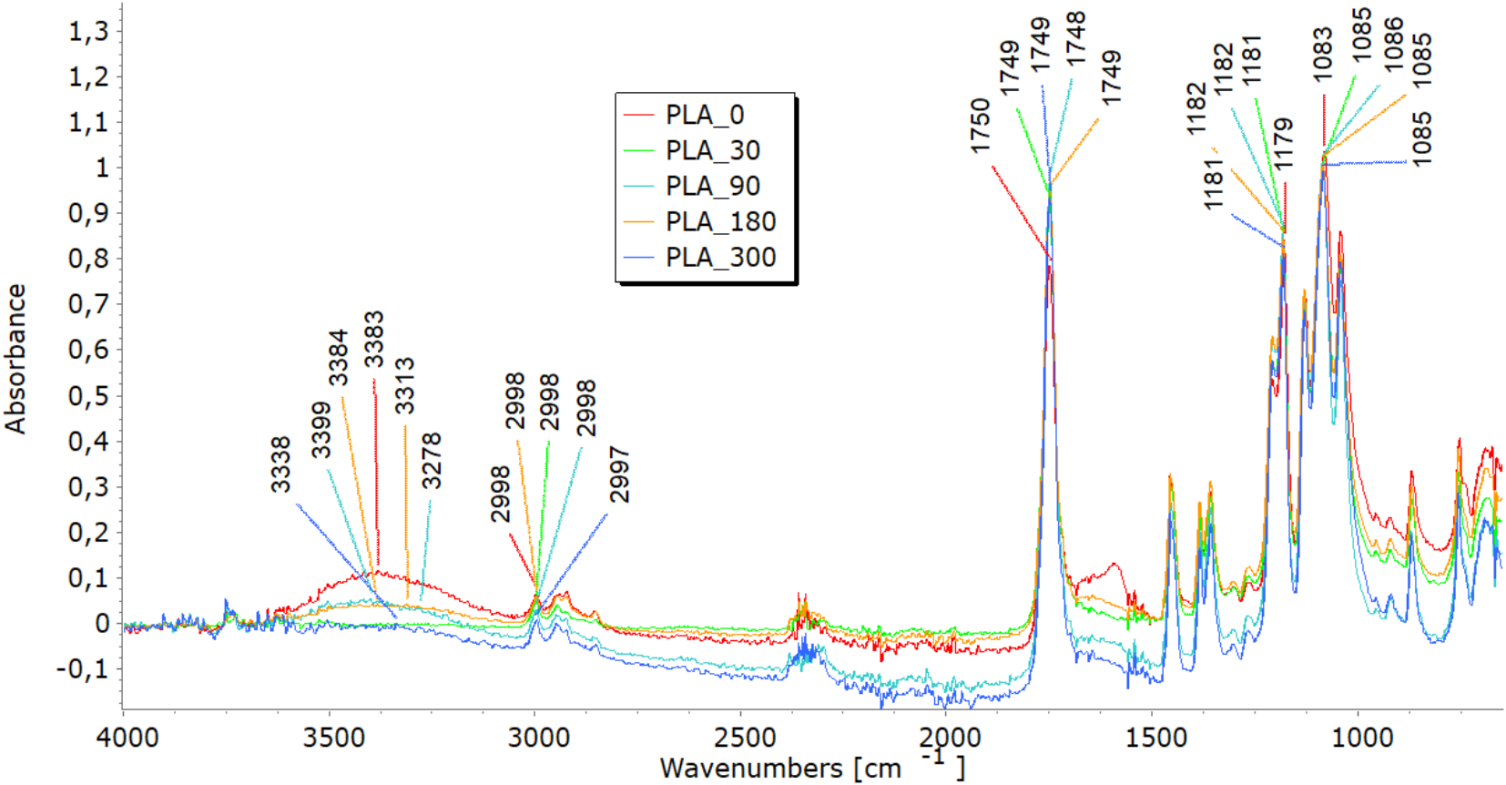
FTIR spectra of unexposed (0) and exposed (30, 90, 180, and 300 days) nonwoven mulches.

FTIR analysis highlighted molecular changes in all fibre types, directly correlating with tensile property trends and structural observations. In the 3600–3200 cm^−1^ spectral range, viscose fibres display a distinct band corresponding to hydroxyl (OH) group stretching vibrations. Bands in the 3000–2800 cm^−1^ range represent carbon-hydrogen (CH) bond stretching, while those in the 1200–1000 cm^−1^ range denote (CO) bond stretching (Fig. 7a). Additionally, peaks at 1470–1420 cm^−1^signify the bending vibrations of CH bonds in cellulose’s aliphatic side chains^26,27^. Variations in band intensity at higher wavenumbers (3333 cm⁻^1^, 2915 cm⁻^1^) indicate modifications in functional groups during degradation, while shifts near 3000 cm⁻^1^ point to changes in the crystalline structure, signalling fibre breakdown. Analysing viscose fibres in nonwoven mulches shows that the band at 2915 cm⁻^1^ remains stable after 30 days. However, further exposure leads to minor changes, including the disappearance of bands at 2848 cm⁻^1^ and 2730 cm⁻^1^. Shifts in band positions within the 1800–600 cm⁻^1^range highlight alterations in hydrogen bonding and changes in dihedral angles at the glycosidic linkage, indicating molecular bond transformations^26^. Bands at 1700–1500 cm^−1^corresponding to carbonyl groups and absorbed water indicate the deterioration of the cellulose structure over time, accompanied by increased water molecule absorption^19,27^. In viscose fibres, degradation was evident through shifts in hydroxyl (OH) and carbonyl (CO) bond peaks, indicating cellulose breakdown and increased water absorption. These molecular changes explain the initial stiffening, reflected in increased breaking force, and the eventual decline in elongation at break as fibres become brittle. The stiffening effect resulting from the material’s shrinkage leads to reduced elongation, supporting the FTIR findings of ongoing cellulose chain breakdown and increased water absorption. SEM images further confirmed extensive surface damage, while WAXD results showed the reorganisation of amorphous regions into crystalline structures, contributing to stiffness before degradation weakened the fibres.

In the FTIR spectra of jute and hemp fibres, characteristic bands corresponding to cellulose are evident, including OH stretching (approximately 3600–3300 cm⁻^1^), CH stretching (around 2900 cm⁻^1^), and CH₂ bending (near 1200 cm⁻^1^)^28–30^, seen in Fig. 7(b and c). The presence and intensity of further bands in the FTIR spectra may be introduced by the hemicellulose and lignin found in jute and hemp fibres. These bands are dependent on the amounts of pectin and lignin present. The characteristic bands of lignin and pectin must be carefully interpreted since they may overlap. The distinctive lignin bands are located at 900–830 cm-1 CH aromatic bending vibrations and in the 1700–1600 cm-1 range, indicating aromatic ring stretching vibrations^28^. OH stretching in the 3600–3200 cm^−1^ range and CO stretching in the 1200–1000 cm^−1^ range may be involved in pectin’s bands. Finding the exact functional groups linked to each band is crucial since, for example, lignin contains aromatic rings that result in peaks linked to aromatic CC bonds. In contrast, pectin has carboxyl groups (CO) that may yield bands in the 1750–1700 cm^−1^range. Lignin typically contains more substantial and pronounced aromatic ring stretching bands than pectin^19^. After exposure to field conditions, FTIR spectra of jute and hemp fibres reveal shifts in characteristic band positions and reduced intensities, notably in OH stretching bands, indicating a transition from crystalline to amorphous cellulose and overall cellulose breakdown. Significant reductions at 1030 cm⁻^1^ and 1745 cm⁻^1^, linked to pectin and lignin, were most notable in jute after 300 days^28–30^. Additionally, impurities from soil exposure detected by FTIR analysis increase some band intensities (around 1700 cm^−1^)^19^. The FTIR analysis reveals molecular changes in hemp and jute fibres, including reductions in cellulose, hemicellulose, lignin, and pectin content. These molecular changes align with SEM observations of surface damage and cracks, reflecting the removal of amorphous components like lignin and pectin. This degradation contributes to mass loss and structural weakening, as seen in decreased tensile strength and elongation at break. The shifts in hydrogen bonding and dihedral angles detected in the FTIR spectra also align with the reduced flexibility and increased brittleness in the nonwoven mulches.

Before exposure, characteristic bands were found in the FTIR analysis of the PLA-fiber-based nonwoven mulch (Fig. 7d). Within the ester linkage of PLA, they included a band at about 3400 cm^−1^ that indicated the stretching vibration of hydroxyl groups (OH) and a band at 1750 cm-1 that indicated the stretching vibration of the carbonyl group (CO). Additionally, bands at around 1180 cm^−1^ corresponded to the stretching vibration of CO bonds and bands at about 3000 cm^−1^ and were linked to the stretching vibration of CH bonds in the PLA’s methylene (CH_3_) groups^21,31–33^. Minimal changes were observed in these distinctive bands when the fibres were exposed to field conditions for 30, 90, 180, and 300 days. This suggests slight changes in the chemical structure but no conclusive evidence of mulch degradation.

The minimal changes in FTIR spectra for PLA fibres, even after prolonged exposure to environmental conditions, correlate with their consistent tensile properties. The breaking force of PLA mulches continues to increase until the end of the experiment, suggesting better resilience to environmental degradation. The stable elongation of PLA mulches is also supported by FTIR results, which demonstrate little to no significant changes in chemical structure.

### Wide-angle X-ray diffraction analyses

**Fig. 8:**
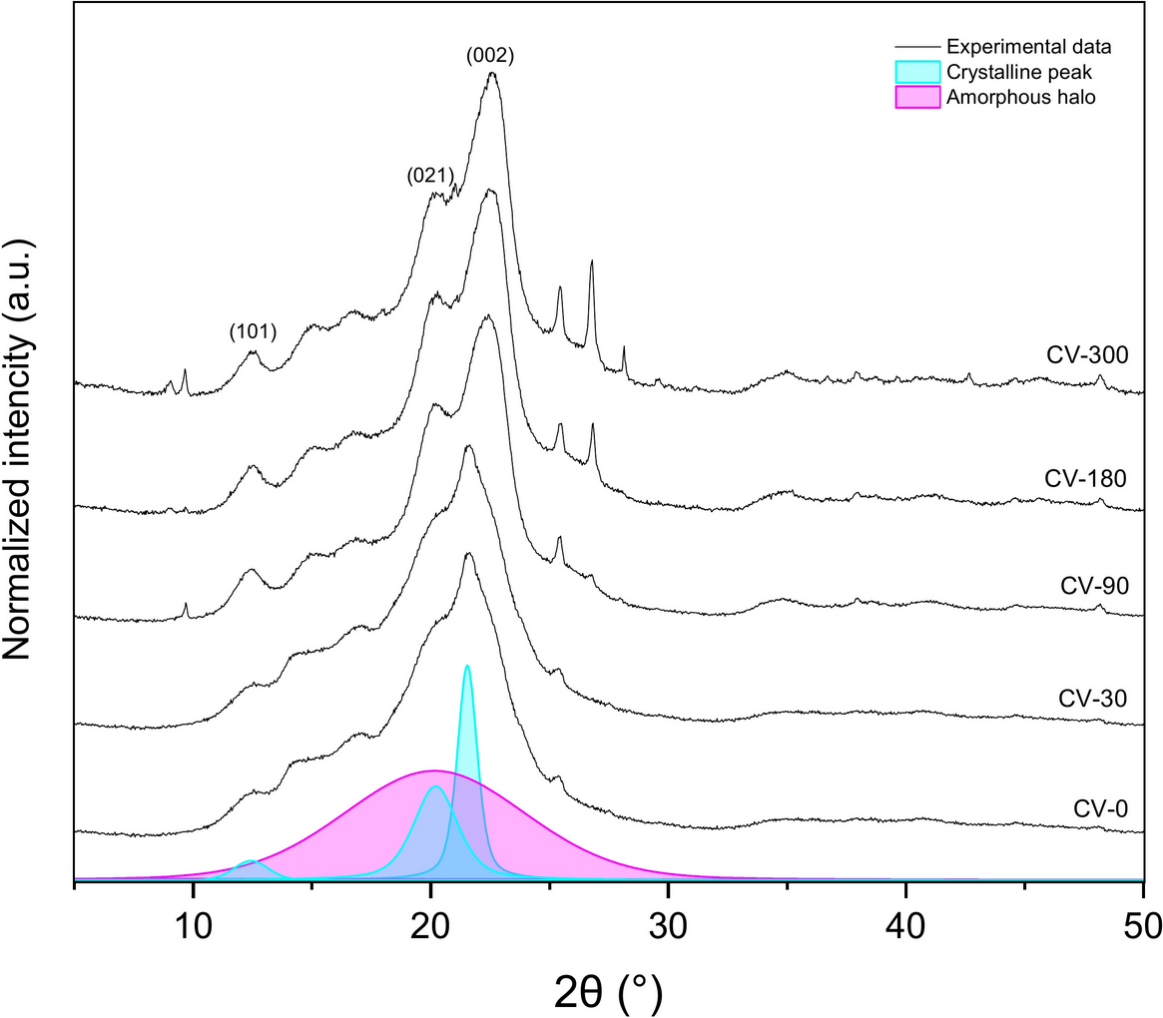
Wide-angle X-ray diffraction diffractograms of regenerated viscose before and after exposure to the field conditions.

The nonwoven samples of regenerated viscose as seen in SEM pictures did contain cotton fibers as well, so diffraction planes of (101) and (021) corresponding to the scattering angle around 2θ = 12.4° and 2θ = 20.2 of cellulose II crystallites are visible on X-Ray diffraction patterns (Fig. 8), respectively with an additional peak of (002) plane at 2θ = 22°^34–37^. After exposure to the field conditions for 90, 180, and 300 days, the crystalline structure of cellulose II is visible, which could be the effect of the degradation of small pieces of cotton fibres.

The lattice length was calculated for the two most intense diffraction peaks corresponding to the (101), (021), and (000) planes. Table 4 lists the changes in the lattice length (d-spacing) of the crystalline forms obtained for different exposure times.

**Table 4:** Wide-angle X-ray diffraction analysis of viscose nonwovens before (0) and during exposure to the field conditions (30, 90, 180 and 300 days).

	**Xc (%)**	**d**_**(101)**_** (nm)**	**d**_**(021)**_** (nm)**	**d**_**(002)**_** (nm)**	**L**_**(101)**_** (nm)**	**L**_**(021)**_** (nm)**	**L**_**(002)**_** (nm)**
CV-0	42.00	0.714	0.440	0.411	5.30	8.16	1.80
CV-30	44.73	0.708	0.433	0.397	7.41	7.70	2.10
CV-90	44.43	0.708	0.440	0.399	7.02	7.34	2.03
CV-180	43.96	0.714	0.438	0.393	8.09	12.86	5.10
CV-300	44.76	0.708	0.442	0.395	9.10	11.09	3.27

Where is Xc—degree of crystallinity; d(hkl)- lattice length (d-spacing); and L(hkl)- average crystalline area.

The degree of crystallinity slightly increases with increasing exposure time, while the lattice length (d-spacing) decreases with exposure time.

Moisture content significantly influences the structural evolution of cellulose in nonwoven mulch fibres, altering lattice parameters and driving longitudinal expansion, transverse contraction, and changes in the monoclinic angle of the cellulose unit cell^38^. In regenerated viscose, the decreasing values of most (hkl) planes over time indicate crystal lattice ordering. This process, facilitated by hydrogen bonding, forms larger crystallites as cellulose chains recrystallise. FTIR analysis supports this, showing shifts in hydroxyl (OH) and carbonyl (CO) functional groups, which reflect ongoing degradation and molecular restructuring. These intramolecular Van der Waals forces arrange the cellulose structure regularly, forming more significant crystalline regions. Increased crystallite size and degree of crystallinity reduce chemical reactivity and water absorption capacity while enhancing the cellulose structure’s physical–mechanical properties, such as stiffness and tensile strength^39^. The gradual increase in crystallinity and crystallite size enhances stiffness and tensile strength by reducing chemical reactivity and water absorption capacity. This is evident in the observed breaking force increase during early exposure, as confirmed by tensile testing. However, as microorganisms preferentially target amorphous regions, which are less ordered and more accessible, these areas become focal points for water interaction. Initially, this leads to higher water absorption in degraded fibres, consistent with FTIR findings of changes in hydrogen bonding. Over time, the reorganisation of cellulose into larger crystalline regions limits water penetration, reducing flexibility and elongation at break. WAXD results complement these observations, showing a correlation between the increasing degree of crystallinity and the stiffening of viscose fibres. This structural evolution, coupled with molecular changes detected in FTIR, explains the initial increase in tensile strength followed by brittleness and reduced pliability. The interplay of moisture-driven lattice reordering, crystallite growth and the degradation of amorphous regions highlights the mechanisms underlying the mechanical and chemical transformations in viscose nonwoven mulches during exposure.

**Fig. 9:**
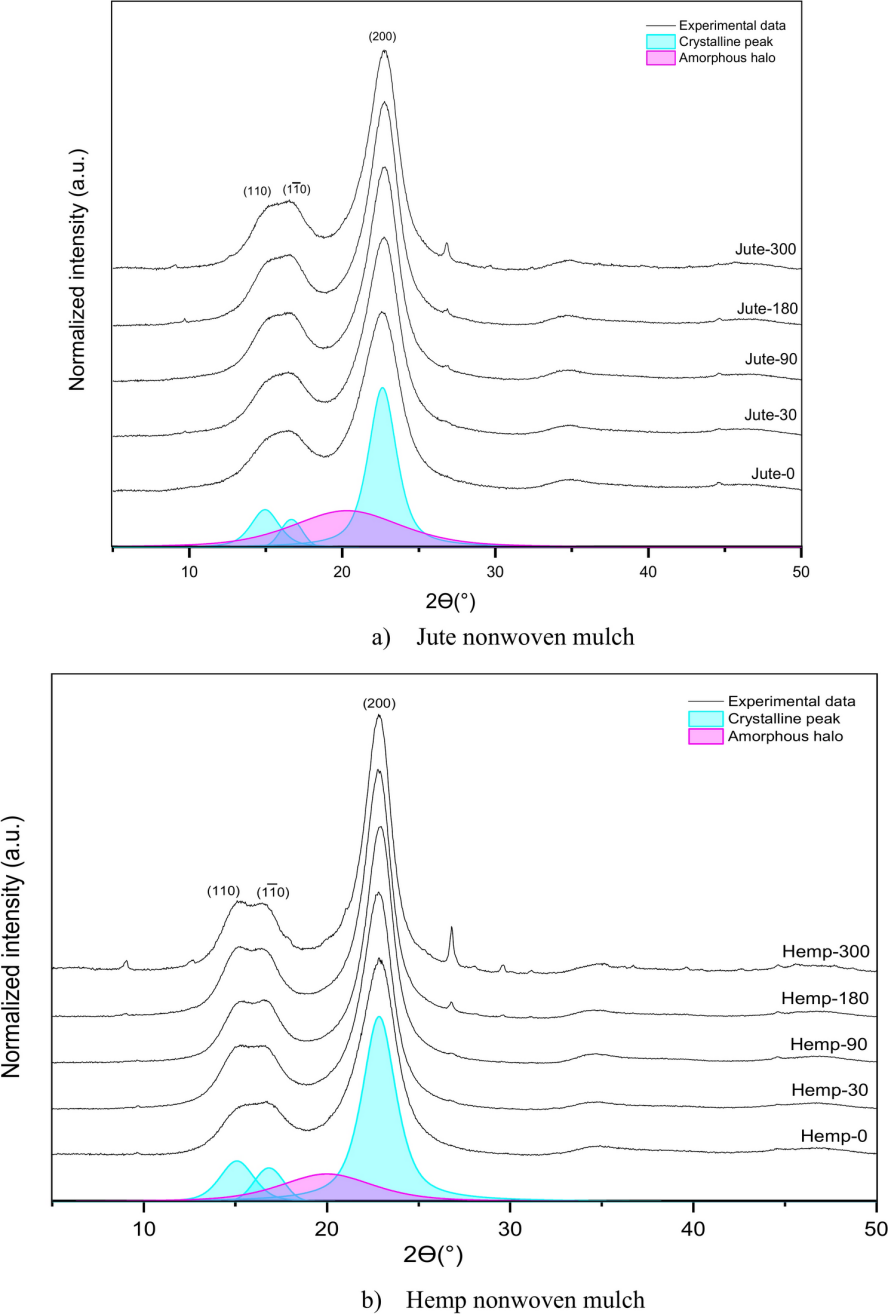
Wide-angle X-ray diffraction diffractograms of (**a**) jute nonwoven mulch and (**b**) hemp nonwoven mulch before and after exposure to the field conditions.

A comparison of the X-ray diffractograms of the studied jute nonwoven samples before and after exposure to the field conditions is shown in Fig. 9a. In the obtained diffractograms, three dominant diffraction peaks were observed at around 2ϴ = 15°, 2ϴ = 17° and 2ϴ = 22.6° corresponding to the (1–10), (110) and (200) cellulose I crystallites planes^40^. The major crystalline peak observed at around 2θ = 22.9° in the X-ray diffractogram of the hemp nonwoven sample (Fig. 9b) corresponds to the cellulose crystallographic plane (200). Other crystallographic planes observed in the diffractogram, namely (1–10) and (110), are obtained at well-defined peaks at 2θ = 15° and 2θ = 17°^41^. The peak around 2ϴ = 26.8° at both samples starts to be visible, i.e. for the sample exposed for 90 days and becomes more profound for the sample after 300 days of exposure, possibly associated with dirt and impurities that samples accumulated during exposure to the open field.

From the presented results in Table 5, it is evident that the degree of crystallinity increases with exposure time, as well as the lattice length (d-spacing) at the characteristic peak 110 and the size of the average crystalline area for the characteristic peak 200 for both jute and hemp nonwoven samples. Additionally, the average size of the crystalline area at the 110 peak for hemp samples also increases with exposure time. The lattice length for the peaks 200 varies and is ultimately smaller than that of the initial jute and hemp samples but with slight differences in values. The same is the case for the average size of the crystalline area at the 110 peak for the jute sample. When the crystalline cellulose content is high, two peaks at (1–10) and (110) crystallographic planes are more pronounced, and when the fibre contains large amounts of amorphous material (such as lignin, hemicelluloses, pectin, and amorphous cellulose)^42^. These peaks are separated, but in the case of jute, they seem more like one broad peak rather than in hemp, probably due to the higher quantity of amorphous materials in the cell walls. Furthermore, that can be seen also in the higher degree of crystallinity in hemp fibre.

**Table 5:** Wide-angle X-ray diffraction analysis of jute and hemp nonwovens before (0) and during exposure to the field conditions (30, 90, 180 and 300 days).

	**Xc (%)**	**d**_**(110)**_** (nm)**	**d**_**(200)**_** (nm)**	**L**_**(110)**_** (nm)**	**L**_**(200)**_** (nm)**
Jute-0	67.12	0.554	0.393	2.35	3.13
Jute-30	68.37	0.554	0.392	2.35	3.41
Jute-90	71.73	0.556	0.391	2.12	3.69
Jute-180	74.82	0.557	0.391	2.35	3.79
Jute-300	71.03	0.557	0.391	2.34	3.74
Hemp-0	82.53	0.551	0.388	2.38	3.77
Hemp −30	86.68	0.557	0.390	2.45	4.75
Hemp −90	86.00	0.561	0.388	2.55	4.36
Hemp-180	89.55	0.568	0.390	2.53	3.92
Hemp-300	88.12	0.572	0.390	2.51	3.94

Where is Xc—degree of crystallinity; d(hkl)—lattice length (d-spacing); and L(hkl)- average crystalline area.

Other literature sources reveal that the changes in the surface crystallinity of cellulose are because of the removal of amorphous components (lignin, pectin, hemicelluloses) resulting from weathering and enhancing the crystallinity degree and crystallite size^43,44^. The remaining constituents are hemicelluloses, pectin substances, or amorphous and para-crystalline regions of the cellulose fibrils. Its mechanical characteristics and overall crystallinity content are predicted to rise if those amorphous polysaccharides break down more quickly than the crystalline cellulose. Furthermore, after the amorphous portion (lignin and hemicellulose) is removed, the semicrystalline component may re-crystallize, which could explain variations in crystallite size^45,46^. The less structured and more readily accessible amorphous regions serve as sites where water interacts with the cellulose structure, as mentioned before. WAXD analysis of jute and hemp nonwoven samples reveals a progressive increase in crystallinity and crystalline area size over time, particularly at the characteristic peaks (110) and (200). The average crystalline area at the 110 peak increases more consistently for hemp fibres. The broad peaks of jute fibres suggest a higher proportion of amorphous components, such as lignin, hemicellulose, and pectin, which initially mask clear crystalline reflections. The observed reduction in lattice length (d-spacing) at the 200 peak indicates tighter packing of cellulose chains, a consequence of the removal of amorphous material during environmental exposure. This process aligns with FTIR findings, which show decreased intensities in OH and CO bands, signalling the breakdown of hemicellulose and pectin.

Removing amorphous regions leads to a stiffer and denser fibre structure, reflected in changes to tensile properties. Initially, the breakdown of amorphous components enhances crystallinity, contributing to a temporary increase in tensile strength. However, as degradation progresses and microbial activity continues to target cellulose, the loss of structural integrity significantly reduces elongation at break. This mechanical weakening corresponds with SEM observations of surface roughness, cracks, and fibre fragmentation, further highlighting nonwoven structures’ progressive breakdown.

**Fig. 10:**
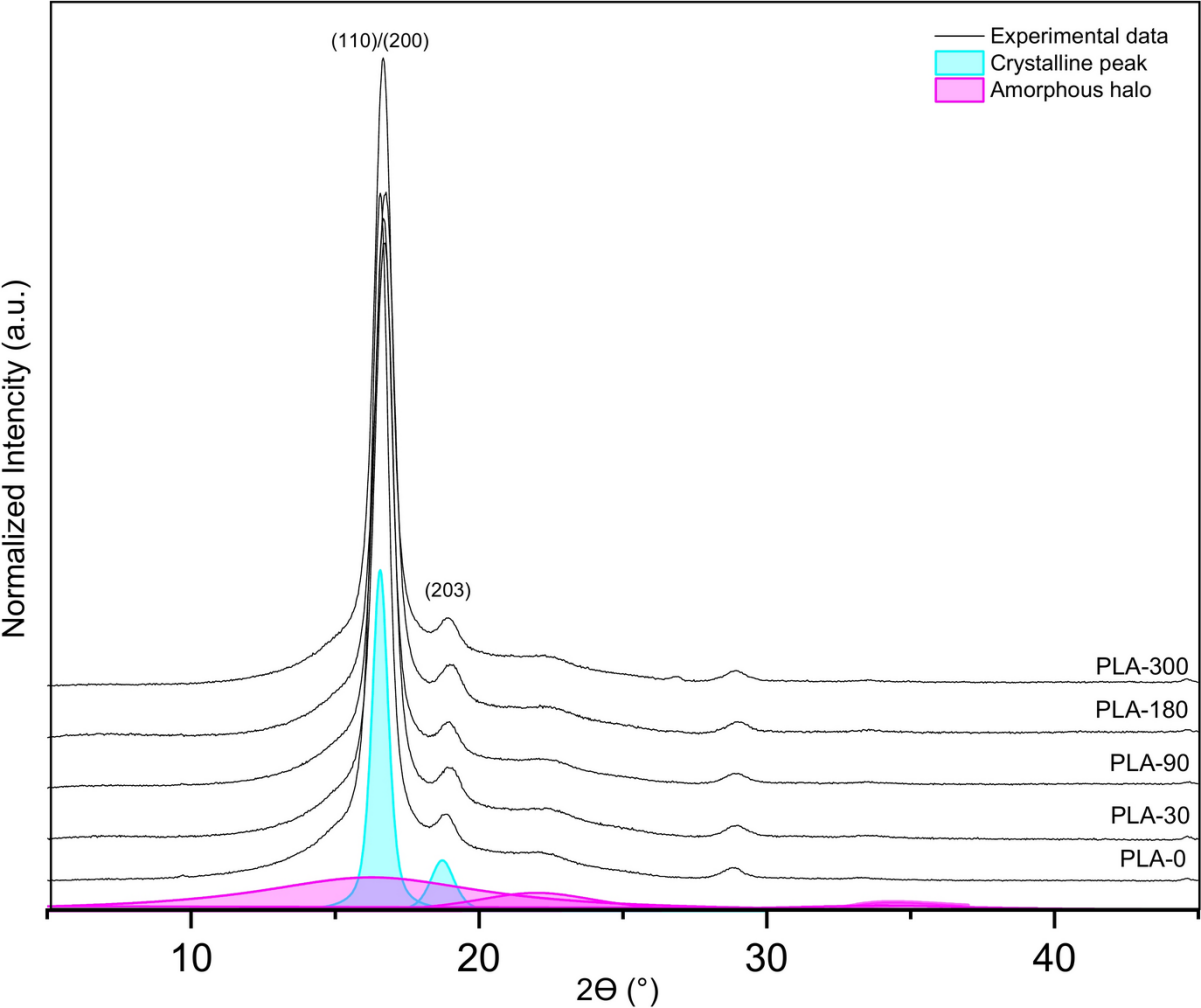
Wide-angle X-ray diffraction diffractograms of polylactide nonwovens before and after exposure to the field conditions.

Figure 10compares the X-ray diffractograms of the PLA nonwoven samples before and after exposure to the field conditions. Two prominent diffraction peaks were seen in all of the acquired diffractograms at about 2ϴ = 16.5° and 2ϴ = 18.8°, which correspond to the (110)/(200) and (203) lattice planes of the α or α’ crystalline phase of PLA. Furthermore, the reflection from the (216) crystallographic planes was attributed to the little diffraction peak at 28.8°^47,48^.

The lattice length was calculated for the two most intense diffraction peaks corresponding to the (110)/(200) and (203) planes. Table 6 lists the changes in the values of d-spacing (lattice length) and crystallinity degree before and after the exposure to the field conditions.

**Table 6:** Results from wide-angle X-ray diffraction analysis of polylactide nonwovens before and after exposure to the field conditions.

	**Xc (%)**	**d**_**(110)/(200)**_** (nm)**	**d**_**(203)**_** (nm)**	**L**_**(110)/(200)**_** (nm)**	**L**_**(203)**_** (nm)**
PLA-0	56.82	0.535	0.470	11.87	12.02
PLA −30	56.05	0.531	0.466	10.42	10.47
PLA −90	56.32	0.532	0.467	11.02	10.75
PLA −180	58.42	0.530	0.465	10.17	11.20
PLA −300	58.69	0.532	0.467	11.37	11.35

Where is Xc—degree of crystallinity; d(hkl)- lattice length (d-spacing); and L(hkl)- average crystalline area.

According to the presented results of the WAXD analysis, there is no significant difference in the value of crystallinity degree between nonwoven PLA-0 mulch and those exposed to field conditions for up to 300 days, which is expected. The initial supramolecular structure of PLA fibres made mulch is ordered, and the α’ crystalline phase of crystallites is visible. The degree of crystallinity slightly increased by the end of the experiment concerning the zero sample. The lattice length was calculated for the most visible peaks, and the average size of the crystalline area slightly decreased with the time of sample exposure. The lower d-spacing values resulted from the degradation circumstances, which allowed the ordering of supramolecular structures and confirmed possible α’ to α phase transition during environmental degradation, which was in-depth testing under laboratory conditions^49,50^. The hydrolytic degradation process of PLA was the reason for the decrease in the average sizes of the crystalline areas, resulting from two phenomena. The first is the perfection of the supramolecular structure, and the second is the erosion of crystallites during degradation. The supramolecular discovery results suggest that a small degree of degradation of PLA materials in crystalline form has occurred, and it has been experimentally demonstrated that much more time must expire under natural conditions before nonwoven PLA mulch achieves significant degradation. The ordering of PLA’s supermolecular structure aligns with the stable tensile properties, as the breaking force continues to increase, while elongation at break remains relatively stable. This suggests that PLA’s resistance to environmental degradation supports its durability and mechanical performance. The minimal changes in PLA’s chemical structure observed through FTIR analysis align with the WAXD findings, supporting PLA’s resistance to degradation and ability to maintain consistent tensile properties.

### Soil temperature and moisture beneath nonwoven mulches

Results of average soil temperature and moisture beneath mulches made of viscose, jute, hemp and PLA fibres, as well as on the control field, spanning from May 2022 to February 2023, are shown in Tables 7 and 8. In May, the temperatures beneath mulches are lower than on the control field, from 0.3 °C to 2.6 °C. The most significant difference is observed beneath viscose and the lowest beneath PLA nonwoven mulch. The temperatures beneath jute and hemp mulches are almost the same. It should be noted that the mulches were laid down on the 5th of May and couldn’t influence the soil temperatures immediately after placement. The temperatures beneath mulches are mostly higher from June to February than on the control field. From July, the temperatures beneath viscose mulches have been lower than beneath other mulch types. The reasons could be found in the highly absorptive viscose fibres that keep the mulches wet (depending on perception) and cool the soil. There are no significant differences in soil temperatures between hemp and jute mulches, except in November, when hemp mulches provide higher soil temperatures. All temperatures beneath mulches were lower in February than on the control field. Although the average air temperature in February was 3.3 °C, there were days when the air temperature went below 0 °C, and the mulches froze, cooling down the soil. Although the mulches, especially cellulose mulches, degrade to some extent during 300 days of exposure, they didn’t change significantly in providing higher temperatures during most of the year. Generally, higher temperatures promote the growth of plants, where the impact of elevated soil temperature should be tested in field conditions during the growing period of particular cultivars to conclude the significance in practical implications.

**Table 7:** The average soil temperature (°C) beneath the mulches and on the control field during the exposure period.

Year	2022								2023	
**Month**	**May**	**June**	**July**	**August**	**September**	**October**	**November**	**December**	**January**	**February**
Air temperature	17.7	22.4	22.9	22.4	15.9	13.0	7.2	3.9	3.7	3.3
CV	16.0	23.5	20.2	19.9	16.8	14.0	11.8	4.7	4.2	3.2
Jute	17.7	23.3	21.7	20.6	17.2	14.1	12.0	4.9	4.4	3.7
Hemp	17.9	22.6	21.5	20.7	17.3	14.3	16.3	5.1	4.5	3.7
PLA	18.3	21.2	20.5	20.3	17.4	14.4	12.7	5.4	4.6	3.8
Control field	18.6	20.4	20.9	20.1	16.6	13.8	11.8	4.8	4.5	4.4

**Table 8:** The average soil moisture (%) beneath the mulches and on the control field during the exposure period.

Year	2022								2023		
**Month**	**May**	**June**	**July**	**August**	**September**	**October**	**November**	**December**		**January**	**February**
RH	93.0	90.0	90.0	91.0	94.0	94.0	96.0	97.0		97.0	96.0
P (%)	53.9	52.0	69.0	22.2	280.0	27.9	119.5	132.3		171.0	27.6
CV	24.1	23.5	17.6	16.8	19.3	23.7	24.3	22.7		20.9	19.9
Jute	23.8	23.3	17.7	15.3	19.0	23.2	23.6	21.9		20.7	17.3
Hemp	23.4	22.6	18.2	15.9	19.4	24.0	23.9	22.5		21.2	18.6
PLA	23.1	21.2	15.5	16.7	18.1	22.6	20.9	21.4		22.4	21.2
Control field	25.1	20.4	13.8	14.5	17.2	20.6	22.1	23.2		24.6	21.8

Where RH is the average relative humidity of air in %, P is precipitation in %.

As was previously mentioned, due to the high absorptivity of viscose mulches, soil temperatures beneath mulches were lower during the exposure period (except in May). A similar pattern is observed in soil moisture (Table 8). From June to December, the viscose nonwoven mulch keeps soil moisture significantly higher than on the control field and beneath other mulch types. The percentage of precipitation influenced soil moisture difference between viscose mulches and the control field, i.e. more perception elevated the soil moisture. Beneath mulches are made of jute, hemp, and PLA fibres, and the moisture was higher than on the control field but not as high as beneath viscose mulches. The soil beneath jute and hemp mulches exhibits slightly higher moisture than the soil beneath PLA mulches and the control field. These findings suggest that certain materials like jute and hemp retain moisture more effectively than mulch made from hydrophobic PLA fibres. The mulches prevent soil drying due to sunlight, providing a better microclimate for growing cultivars. From December to February, soil moisture was lower in winter under all mulches.

### Soil microorganisms

When exposed to field conditions, natural-based mulches degrade, providing a food source for soil bacteria and fungi. The soil pH mainly impacts bacteria richness, diversity and abundance, while soil nutrients impact fungal richness, diversity, and abundance. Bacterial diversity first increases with some degree of organic matter degradation, reaching a maximum diversity at a moderate degradation level and then decreasing as organic matter degradation completes. Generally, fungi are more sensitive to organic matter degradation than bacteria. Most fungal phyla show a decrease in relative abundance with increased degradation of organic matter^51,52^. Bacterial activity in soil is typically higher than fungal activity, which is observed by higher calorimetric values of bacteria^49^. While fungi are essential for breaking down the outer protective layer of cellulose fibre, bacteria are responsible for the degradation of complex organic material like cellulose. The number of bacterial and fungus colonies can vary based on soil amendments and environmental conditions. The diversity and abundance of bacterial and fungal colonies reflect soil health and, therefore, organic matter degradation, with bacteria generally being more active in response to organic amendments and fungi being more sensitive to organic degradation^53–55^. Considering the theory of microorganism behaviour in the soil during organic matter degradation, after 300 days of exposure, samples of soil beneath each nonwoven mulch type were taken and tested (Table 9).

**Table 9:** Number of microorganisms (bacterial and fungi) colonies in the soil beneath the nonwoven mulches and control field after 300 days of exposure to the field conditions.

Sample	Number of bacterial colonies (CFU/ml)	Number of bacterial fungi (CFU/ml)
CV	3.64 × 10^7^	4.43 × 10^4^
Jute	3.11 × 10^7^	2.57 × 10^4^
Hemp	2.84 × 10^7^	5.80 × 10^4^
PLA	1.15 × 10^7^	6.53 × 10^4^
Control field	3.75 × 10^7^	4.23 × 10^4^

After 300 days of exposure, jute and hemp mulches degraded into small pieces, where big enough samples for testing weren’t possible to collect. Generally, degradation of bast fibres starts with fungi attacking the protective fibre layer (lignin and hemicellulose), which allows penetration of bacteria inside the fibre where cellulose is food for bacteria. Due to available food sources, the number of bacterial colonies increases until they peak, followed by a bacterial decrease as food sources decrease. At the same time, as the degradation process reaches the peak, the number of fungi colonising starts to decline.

Considering the mechanism of bast fibre degradation and soil microorganisms beneath jute and hemp mulches compared to the control field, it can be concluded that the outer layer of hemp fibres still feeds the fungus colonies, and bacteria will enter the fibre to degrade cellulose. On the contrary, the outer layer of jute fibres is mostly eaten by fungus, and bacterial colonies will start to eat the cellulose. Therefore, the nonwoven mulch structure deteriorated due to field conditions, but fibres within the nonwoven structure are just about to reach the degradation peak. The tensile properties of jute and hemp mulch decrease due to structure deterioration and specific decomposition of the fibres.

The soil beneath viscose mulches has bacterial and fungal colonise counts similar to those on the control field. The degradation of mulches is influenced by the balanced presence of both bacterial and fungal colonies, i.e., the degradation process is about to peak. Considering that viscose mulches change structure from soft and fluffy to condensed and paper-like, viscose fibres FTIR analysis indicates significant degradation over time while tensile properties significantly decrease, it can be concluded that viscose fibres degrade simultaneously with the deterioration of the structure.

The mechanism of PLA fibre degradation is different from cellulose fibre degradation. The biodegradation of PLA fibres begins with hydrolysis, where water molecules break down the ester bonds in the PLA polymer chains. This step doesn’t directly involve microorganisms but is essential to make the polymer more accessible to them. Hydrolysis reduces PLA’s long polymer chains into shorter chains and oligomers, which are more susceptible to microbial attack. Microorganisms colonise the surface of the PLA fibres and start producing enzymes (proteases, lipases, and esterases) that facilitate further breakdown of oligomers and shorter polymer chains into even smaller molecules.

Therefore, compared with the control field, the number of bacterial colonies in the soil beneath PLA nonwoven mulch is significantly lower, while the number of fungi increased. This shows that fibre degradation begins, meaning the water molecules (from the environment, i.e. participation) break down ester bonds, allowing the attachment of fungus to the outer layer of the fibre. Since the degradation just started, the bacterial colony number is suppressed. SEM, FTIR, and WAXD analyses confirm the beginning of PLA fibre degradation.

## Conclusion

Nonwoven mulches made from jute, hemp, viscose, and PLA fibres undergo significant physical, chemical, and structural changes when exposed to environmental conditions such as soil, sunlight, and precipitation over 300 days. Visually, all mulches show discolouration and deterioration, particularly mulches made from jute and hemp fibres, due to the fibre’s lignin degradation under UV radiation, which leads to fibre yellowing and fading. Dimensional changes, including shrinkage and expansion, are observed, especially in viscose mulches, where soft mulch structures become compact, dense and paper-like. Soil particles, plant residues, and fibre arrangement further influence fluctuations in mass per unit area and variations in thickness besides mulch compaction. SEM analysis reveals surface cracks, roughness, and structural disintegration, particularly in cellulose-based fibres, confirming that microbial activity targets amorphous regions. Tensile properties reflect these structural changes, with an initial increase in breaking force due to fibre densification and crystalline reorganisation. Viscose mulches show the highest breaking force increase, followed by PLA and hemp mulches. Over time, microbial activity accelerates degradation in jute and hemp mulches to the point that after 300 days, it was impossible to collect samples from the field and, thus, to conduct tensile tests. Viscose mulch’s tensile properties decline due to fibre surface damage and cellulose breakdown. FTIR analysis confirms the breakdown of cellulose, lignin, and hemicellulose, while WAXD highlights increased crystallinity and crystalline area size as amorphous components are removed. This reorganisation initially enhances strength but ultimately reduces elongation and flexibility as degradation progresses.

PLA shows only minor structural changes, consistent with its slower hydrolysis and maintained tensile properties. Due to their stable crystalline structure, the PLA mulches retain breaking force and elongation at the break, as confirmed by minimal FTIR and WAXD changes.

The degradation of tested mulches occurs on two levels: structural changes in the nonwoven fabric and molecular transformations in the fibres. Structural mulch changes dominate early exposure, while FTIR and WAXD reveal significant molecular degradation in later stages. The placement of tested mulches on the soil influences soil conditions. Viscose mulches enhance soil moisture while jute and hemp mulches increase microbial activity. Mulches made from PLA fibres show lower bacterial activity but increased fungal colonisation over time.

This study provides a comprehensive understanding of the biodegradation mechanisms of cellulose and PLA nonwoven mulches, connecting their physical, chemical, and mechanical changes. The findings highlight the interplay between fibre composition, environmental factors, and microbial activity, offering valuable insights for developing sustainable mulching materials in agriculture.

## Data Availability

The authors declare that the data supporting the findings of this study are available within the paper. Any raw data will be made available from the corresponding author upon reasonable request.
